# Comparison of diabetes management in five countries for general and indigenous populations: an internet-based review

**DOI:** 10.1186/1472-6963-10-169

**Published:** 2010-06-17

**Authors:** Damin Si, Ross Bailie, Zhiqiang Wang, Tarun Weeramanthri

**Affiliations:** 1 Centre for Chronic Disease, School of Medicine, University of Queensland, Brisbane, Australia; 2 Menzies School of Health Research, Charles Darwin University, Darwin, Australia; 3 Western Australia Department of Health, Perth, Australia

## Abstract

**Background:**

The diabetes epidemic is associated with huge human and economic costs, with some groups, such as indigenous populations in industrialised countries, being at especially high risk. Monitoring and improving diabetes care at a population level are important to reduce diabetes-related morbidity and mortality. A set of diabetes indicators has been developed collaboratively among the Organisation for Economic Co-operation and Development (OECD) countries to monitor performance of diabetes care. The aim of this review was to provide an overview of diabetes management in five selected OECD countries (Australia, Canada, New Zealand, the US and the UK), based on data available for general and indigenous populations where appropriate.

**Methods:**

We searched websites of health departments and leading national organisations related to diabetes care in each of the five countries to identify publicly released reports relevant to diabetes care. We collected data relevant to 6 OECD diabetes indicators on processes of diabetes care (annual HbA1c testing, lipid testing, renal function screening and eye examination) and proximal outcomes (HbA1c and lipid control).

**Results:**

Data were drawn from 29 websites, with 14 reports and 13 associated data sources included in this review. Australia, New Zealand, the US and the UK had national data available to construct most of the 6 OECD diabetes indicators, but Canadian data were limited to two indicators. New Zealand and the US had national level diabetes care data for indigenous populations, showing relatively poorer care among these groups when compared with general populations. The US and UK performed well across the four process indicators when compared with Australia and New Zealand. For example, annual HbA1c testing and lipid testing were delivered to 70-80% of patients in the US and UK; the corresponding figures for Australia and New Zealand were 50-60%. Regarding proximal outcomes, HbA1c control for patients in Australia and New Zealand tended to be relatively better than patients in the US and UK.

**Conclusions:**

Substantial efforts have been made in the five countries to develop routine data collection systems to monitor performance of diabetes management. Available performance data identify considerable gaps in clinical care of diabetes across countries. Policy makers and health service providers across countries can learn from each other to improve data collection and delivery of diabetes care at the population level.

## Background

### Magnitude of the diabetes epidemic in five selected countries

Diabetes mellitus is a significant health problem in many countries, including Australia, Canada, New Zealand, the United Kingdom (UK), and the United States (US). Table 1 shows the number of people affected by diabetes, deaths due to diabetes, and estimated economic costs in each of these five countries [1-7]. Absolute numbers of people with diabetes (diagnosed and undiagnosed) varied greatly from country to country. However, estimated crude prevalence rates were consistently around 4%-6%, and estimated crude case fatality ratios were between 2.5/1000 and 3.8/1000 per year (these may not reflect the quality of diabetes control across the nations as data collection systems were different).

**Table 1:** Diabetes prevalence, deaths due to diabetes, and economic costs in five selected countries*

Country	Population^† ^(million)	People with diabetes (million)	Crude prevalence	No. of deaths due to diabetes		Crude case fatality ratio^‡ ^(n/1000)	Costs/year (billion)
						
				Primary cause	Associated cause		
Australia [1,2]	19.9	0.9	4.5%	3,329	8,138	3.7	AU$ 0.68
Canada [3]	32.5	1.7	5.2%	6,137	-	3.6	CA$ 1.60
New Zealand [4]	3.9	0.18	4.6%	802	-	4.5	NZ$ 0.18
UK [5]	60.2	2.8	4.7%	7,000	26,000	2.5	£ 3.50
US [6]	293.0	18.2	6.2%	69,308	143,754	3.8	US$ 132

* Data in the table refer to a period between 2002 and 2004.

† Populations were obtained from the International Data Base, US Census Bureau [7].

‡ Based on diabetes as primary cause of death.

It is predicted that the number of people worldwide with diabetes will double in the next generation [8]. As well as the suffering of individuals and their families, diabetes poses a huge economic burden to nations' health care systems, mostly due to expenditures relating to long term diabetes complications and hospitalisations. In the UK, it is estimated that the National Health Services expenditure on diabetes will account for ten percent of its total annual budget by 2011, double the level in 2004 (see Table 1).

Due to such huge human and economic costs, nations have intensified their efforts to combat diabetes. In Australia, the National Diabetes Strategy was implemented in 1999, following the designation of diabetes as a National Health Priority Area in 1996 [9]. The Canadian Diabetes Strategy was initiated in 1999 with substantial investments to establish a national partnership for effective prevention and control of diabetes [10]. Diabetes was identified as one of the 13 Priority Population Health Objectives in New Zealand in 2000, in an effort to reduce the impact of diabetes and population health inequalities [11]. In the US, the National Diabetes Education Program was launched in 1997 to improve prevention and management of diabetes [12]. In the UK, a National Service Framework for Diabetes was developed in 2003 with explicit care standards and delivery strategies [13].

### Diabetes epidemic among indigenous populations

Diabetes prevalence rates are much higher in indigenous populations of high income ex-colonial countries than in people of European origin. On average, American Indians and Alaskan Natives are 2.3 times as likely to have diabetes as non-Hispanic whites of similar age in the US [6]. In Canada, prevalence of diabetes among First Nations peoples is at least three times the national average [3], and First Nations peoples with diabetes are 3-4 times more likely to suffer from heart disease and stroke. Maori and Pacific Island people in New Zealand have nearly three times higher prevalence of diabetes, and their mortality rates from diabetes in the 40-65 age group are nearly ten times higher than for Europeans [4]. While Aboriginal and Torres Strait Islander people in Australia experience three to four times higher prevalence of diabetes than in the general population [14], their death rate associated with diabetes for the 35-54 years group is 27-35 times that of non-Indigenous Australians [15].

Despite their diverse geographical locations, cultures and traditions, the epidemic of diabetes among indigenous peoples is largely driven by rapid social and environmental changes which aggravate preventable risk factors such as unhealthy diet, decreased physical activity and tobacco use [16]. The root causes of these lifestyle-related risk factors are the long history of dispossession, exclusion, discrimination and associated social and economic disadvantage (including poor income, education, employment and living conditions) among indigenous populations [17].

### Health systems performance for diabetes care

There is a renewed interest in developing and using quality indicators to measure and benchmark the performance of health care systems with regard to diabetes care. For example, National Health Priority Area diabetes indicators have been employed to report national progress on prevention and control of diabetes in Australia. Available data relating to such indicators were published in 1998, 2002 and 2008 [1,18,19]. The US annual National Healthcare Quality Report uses a set of 12 indicators to assess national performance in diabetes management [20]. Likewise, the US National Committee for Quality Assurance uses the Comprehensive Diabetes Care measure to assess the quality of diabetes services provided by managed care plans (commercial insurance, Medicare and Medicaid) [21]. Due to the diversity of the indictors across countries, it has been difficult to conduct an international comparison of diabetes care.

Efforts in the development, specification, and field-testing of measures for diabetes care have been carried out in the form of international collaborations. The European Union Diabetes Indicators Project was conducted during 2000-2002 and a set of core and secondary indicators was proposed to monitor diabetes and its complications in European Union/European Free Trade Area countries [22]. An Organisation for Economic Co-operation and Development (OECD) health technical paper delineates recommended indicators for the quality of diabetes care at the health system level in OECD countries [23]. These indicators were selected using criteria assessing impact on health, policy importance, susceptibility to being influenced by the health care system, and feasibility of data collection. As Australia, Canada, New Zealand, the US, and the UK are all participating in the indicator development and will be the future users of such measures, it is useful to assess the availability of data derived from current systems to provide information for the indicators.

### Objectives of this review

(1) To assess the availability and quality of data which can be used for constructing OECD diabetes indicators in five selected countries (Australia, Canada, New Zealand, the US, and the UK);

(2) To compare the quality of diabetes management at the national level among these five countries using OECD indicators; and

(3) To compare the quality of diabetes management among indigenous populations in Australia, Canada, New Zealand, and the US using these indicators.

Reasons for selecting these five countries in this review include: 1) they are all OECD countries with advanced economies; 2) indigenous health is an important issue in Australia, Canada, New Zealand and the US; and 3) while the UK is not related to the indigenous health issue, it has a health care system similar to those in Australia, Canada and New Zealand.

## Methods

### Search strategy for identification of data

A. Internet-based reports. As national level delivery of services and quality of care data are usually published in monographs or reports by health departments of national governments, websites of the five countries' health departments were first searched to identify publications related to diabetes care. Then, the websites of the leading national organisations relating to diabetes care in each country were also searched to obtain relevant reports. A list of internet sources for data searching is shown in Table 2. The following terms were used for the search: diabetes and (family practice, general practice, primary care, outpatients, quality, audit, guideline adherence, quality indicators, performance) (terms in the brackets connected by or). The search was initially conducted in May 2006 and updated in August 2009/February 2010. We included reports released during the period January 2000 - July 2009. Only English reports were included in this review.

**Table 2:** Internet sources for identification of diabetes-related national reports in five selected countries

Country/Organisation	Internet address
**Australia**	
Department of Health and Ageing	http://www.health.gov.au
Australian Institute of Health and Welfare (AIHW)	http://www.aihw.gov.au/
Health*Insite*	http://www.healthinsite.gov.au/topics/Diabetes
Australian Indigenous Health*InfoNet*	http://www.healthinfonet.ecu.edu.au/
Australian Divisions of General Practice (ADGP)	http://www.adgp.com.au/site/index.cfm
Primary Health Care Research & Information Service	http://www.phcris.org.au/
Diabetes Australia	http://www.diabetesaustralia.com.au/
Australian Diabetes Society	http://www.diabetessociety.com.au/
International Diabetes Institute	http://www.idi.org.au/home.htm
**Canada**	
Health Canada	http://www.hc-sc.gc.ca/english/index.html
Canadian Institutes of Health Research	http://www.cihr-irsc.gc.ca
Canadian Diabetes Association	http://www.diabetes.ca/
National Aboriginal Diabetes Association	http://www.nada.ca/
Statistics Canada	http://www.statcan.gc.ca/
**New Zealand**	
Ministry of Health	http://www.moh.govt.nz/moh.nsf
New Zealand Health Information Service	http://www.nzhis.govt.nz
Diabetes New Zealand	http://www.diabetes.org.nz/
**The UK**	
Department of Health	http://www.dh.gov.uk/Home/fs/en
Diabetes UK	http://www.diabetes.org.uk/
National Electronic Library for Health	http://www.library.nhs.uk/
Audit Commission	http://www.audit-commission.gov.uk/health/
**The US**	
Department of Health and Human Services	http://www.hhs.gov
Centres for Disease Control and Prevention (CDC)	http://www.cdc.gov/diabetes/
Agency for Healthcare Research and Quality (AHRQ)	http://www.ahrq.gov
Indian Health Service (IHS)	http://www.ihs.gov
Office of Minority Health Resource Center	http://www.omhrc.gov
National Committee for Quality Assurance (NCQA)	http://www.ncqa.org
American Diabetes Association (ADA)	http://www.diabetes.org
National Institute of Diabetes, Digestive and Kidney Diseases (NIDDK) of the National Institutes of Health (NIH)	http://www.niddk.nih.gov

B. The reference list of each retrieved report was also scanned to identify relevant information sources.

### Types of outcome measures

The OECD diabetes indicators were used to assess the quality of diabetes management [23]. These nine indicators cover clinical processes of diabetes care as well as proximal and distal outcomes (Table 3). Notably, for proximal outcomes, no specific cut-points for HbA1c and LDL cholesterol have been recommended by the indicator developers, reflecting a lack of international consensus on the threshold levels of these outcome indicators. The use of the six process and proximal outcome indicators was based on robust evidence and international consensus [23]. The use of the distal outcome indicators have not been universally accepted due to operational concerns. However, these distal outcomes represent long term outcomes of diabetes care, providing insight into the long term outcomes of overall health care systems.

**Table 3:** OECD diabetes indicators

Area	Indicator name (number)
Processes of diabetes care	(1) Annual HbA1c testing
	(2) Annual LDL cholesterol testing
	(3) Annual screening for nephropathy
	(4) Annual eye examination
Proximal outcomes	(5) HbA1c control
	(6) LDL cholesterol control
Distal outcomes	(7) Lower extremity amputation rates
	(8) Kidney disease in persons with diabetes
	(9) Cardiovascular mortality in patients with diabetes

We initially attempted to retrieve relevant information from selected countries to construct all of these nine indicators. However, we found that data for the three distal outcome indicators were rarely available at the national level, mainly due to difficulties in obtaining relevant information on denominators (all patients with a diagnosis of diabetes) for these indicators. Therefore, this review will only report results regarding the six process and proximal outcome indicators.

### Inclusion criteria

#### Types of data sources

Two types of data sources were included: routine data collection systems in health care; and cross-sectional studies (surveys). Cross-sectional studies or surveys are generally considered to be suitable designs for evaluating quality of health care [24].

#### Types of populations

'Study' populations were required to meet the following three criteria:

1. With diagnosis of type 1 or type 2 diabetes;

2. Aged 16 years or more;

3. Drawn at the national level (e.g. from multiple provinces/states/regions).

Data that focused on gestational diabetes were excluded.

#### Outcome measures

Data included were related to one or more of the six OECD process and proximal outcome indicators as specified in Table 3.

### Criteria for assessing data quality

We adapted a quality assessment tool for observational studies developed by Wong et al [25]. The adapted tool measured data quality in terms of sample representativeness, national coverage of data, measurement objectiveness and response rate (see Additional file 1). An overall quality assessment score is calculated to rate the data quality as poor (0-0.33), satisfactory (0.34-0.66) or good (0.67-1).

### Review process and data abstraction

Initially, all reports/papers identified by the internet search were screened by the reviewer (DS) against the inclusion criteria, to identify potential reports/papers which merited full-text reviews. A second reviewer (ZW) repeated this process independently six months later for selection of reports/papers into the review. The full reports/papers that were identified as possibly meeting the inclusion criteria by either reviewer were retrieved for further assessment.

At the full-text level, a standardised abstraction form was used independently by each reviewer to extract information on data sources, targeted populations, outcomes, and data quality. Any discrepancies of results between the reviewers were resolved through discussion and consensus.

When the same data sources provided multiple year reports, all relevant reports were reviewed. However, only the data from the most recent report (or from years 2005-2007 which most countries had data available) were included in this review.

In this paper, unless otherwise specified, the term "patients" refers to people diagnosed as having diabetes.

## Results

**Figure 1:**
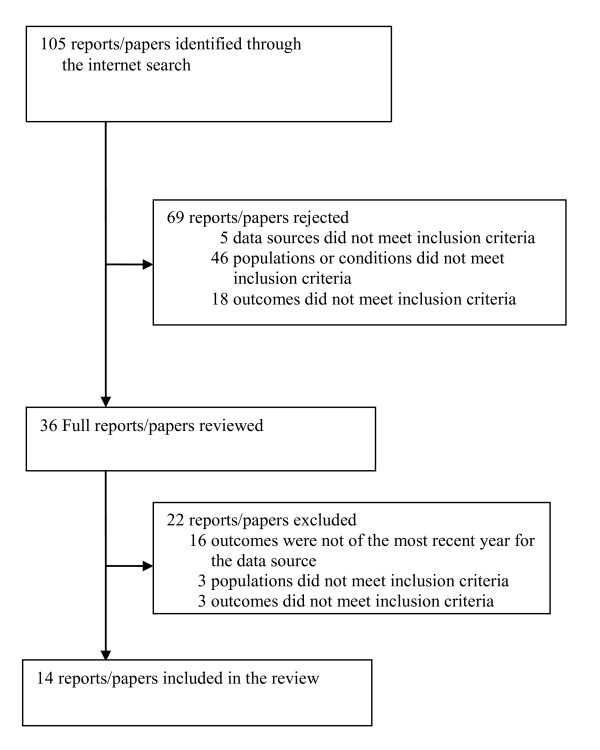
**Search results**.

One hundred and five reports/papers were initially identified by the internet search of 29 websites as specified in Table 2) (Figure 1). Of those reports/papers, 36 met our explicit inclusion criteria and their full-texts were retrieved for data abstraction. During the full-text review, 22 reports/papers failed to meet inclusion criteria, and the remaining 14 were included for the current review [1,20,26-37].

### Summary of identified data sources

The 14 reports related to 13 data sources which provided information relevant to the 6 OECD diabetes indicators (Table 4). These data sources can be broadly categorised into three types: 1) medical record data - for example, the General Practice Divisions Information Online System in Australia, the Health Plan Employer Data and Information Set in the US, and the National Diabetes Audit in the UK; 2) administrative health insurance data, such as the Health Insurance Commission General Practice Statistics in Australia; and 3) population-based survey data - for example, the Canadian Community Health Survey.

**Table 4:** Summary of identified data sources for constructing OECD diabetes indicators

Country	Data source/year	Data collection method/frequency	Availability of data for 6 OECD indicators
**Australia**	General Practice Divisions Information Online System 2006-07 [26]	General Practice Divisions network includes about 120 regionally based divisions which facilitate and support active participation by GPs and general practices in primary care activities and programs. General practices' membership with regional divisions is voluntary. All Divisions network members are required to report against a set of National Performance Indicators (including 9 diabetes care related indicators). Diabetes care data are collected annually from practice level diabetes registers.	Indicators 1, 2, 5,6

	National General Practice Divisions Diabetes Program, 2002 [35,36]	The National General Practice Divisions Diabetes Program collected diabetes care data from 16 divisions who used the same electronic diabetes patient register (CARDIAB). GPs provided patient data for entry into divisional registers. Data were extracted from registers only for three years (2000, 2001 and 2002). The project was one-off, with no ongoing data collection arrangement.	Indicators 1-6

	Australian National Diabetes Information Audit & Benchmarking 2006 [27]	Diabetes specialist services are delivered primarily through over 60 Diabetes Centres across the nation, and relevant data are collected biennially by the National Association of Diabetes Centres through the Australian National Diabetes Information Audit and Benchmarking (ANDIAB) program. In 2006, 15 Diabetes Centres and 1 specialist endocrinologist in private practice provided de-identified data on a total of 1624 individuals seen during the one-month survey period of October (or November) 2006.	Indicators 1-6

	Health Insurance Commission General Practice Statistics, 1999-2000 [1]	Australia has publicly funded, universal health insurance - Medicare. The computerised Medicare billing database records occasions of services provided by general practitioners and specialists. It contains information on service utilisation (e.g. laboratory investigations) for people with diabetes. However, the use of Medicare data to monitor diabetes care at the national level is on an ad hoc basis.	Indicators 1-4

	The Australian Diabetes, Obesity, and Lifestyle Study (AusDiab), 1999-2000 [34,37]	The AusDiab was a population-based study of 11,247 people from randomly selected areas of Australia in 1999-2000. Data collection methods included face-to-face interviews and physical and laboratory measurements. Data related to diabetes care were based on 439 participants who had previously diagnosed type 2 diabetes. The study was a one-off national survey.	Indicators 4-6

**Canada**	Canadian Community Health Survey 2005 [28]	A national, population-level periodical survey operated on a two-year data collection cycle, which includes diabetes-specific components (optional for inclusion at a province/territory level) in the questionnaire to collect data on diabetes care for those with self-reported diabetes in the general population. The 2005 survey only collected diabetes care related data from six out of thirteen Canadian provinces/territories.	Indicators 1, 4

**New Zealand**	National Get Checked Programme 2006 [29]	The nationally funded programme provides a free annual check for all people with a diagnosis of type 1 or type 2 diabetes. Services are delivered at the primary level by general practitioners or trained primary care nurses, and a particular effort has been made to ensure maximum access by Maori and Pacific Island peoples. Data are then passed to the Primary Health Organisations, which maintain registers for free annual checks and report aggregated datasets to the Local Diabetes Teams. Local Diabetes Teams combine data and provide annual reports to the District Health Boards and the Ministry of Health on an annual basis.	Indicators 1-6

**The US**	Medical Expenditure Panel Survey (MEPS), 2004 [30]	The MEPS has two components: the household component and the insurance components. On an annual basis, the household component collects data from a nationally representative sample of families and individuals through household interviews. Information collected includes demographic characteristics, health conditions, health status, use of medical services, charges and source of payments, etc.	Indicators 1, 4

	CDC Behavioural Risk Factor Surveillance System (BRFSS) [20]	The BRFSS is a state-based system of health surveys that collects information on health risk behaviours, preventive health practices, and health care access related to chronic disease and injury. Data are collected each year in all 50 states and the District of Columbia, through telephone interviewing of a representative sample of more than 350,000 adults.	Indicators 1, 4

	National Health and Nutrition Examination Survey (NHANES), 1999-2004 [30]	The NHANES collects information every few years on a national sample of approximately 40,000 people using face-to-face interviews and physical and laboratory measurements. It asks participants whether they have a history of diabetes and performs blood analyses. The NHANES is valuable in generating national prevalence estimates for diabetes (diagnosed and undiagnosed) as well as in assessing cardiometabolic control among patients.	Indicators 5, 6

	Health Plan Employer Data and Information Set (HEDIS), 2006 [31]	The HEDIS is a standardised tool used by the National Committee for Quality Assurance (NCQA) to collect performance data for managed care organisations. HEDIS data cover people enrolled in managed-care plans (commercial insurance, Medicare, and Medicaid). Commercial insurance is usually paid by employers for their employees, Medicaid covers certain individuals and families with low incomes, and Medicare is available for people 65 years or older as well as certain people with disabilities. Managed care organisations are the main source of health care services for persons with diabetes in the US. Data are collected annually by auditing clinical records.	Indicators 1-6

	Indian Health Service, Clinical Reporting System, 2006 [32]	The Indian Health Service (IHS) is an agency within the US Department of Health and Human Services, providing health care services to eligible American Indian and Alaska Native people. The IHS reports to Congress each year on the quality of health care provided to its patients as required by the Government Performance and Results Act (GPRA). The GPRA measures comprise a set of clinical and non-clinical indicators, including 6 indicators related to diabetes management. Data for the GPRA diabetes indicators are obtained through the IHS Clinical Reporting System which extracts information from individual patient health records at participating health facilities on an annual basis.	Indicators 1-6

**The UK**	National Diabetes Audit 2005-2006 [33]	The UK has established the National Clinical Audit Support Programme to assess current diabetes care at primary and secondary care sectors, and to review progress towards achieving the standards set out in the National Service Framework. In 2005-2006, 43% (131/305) of Primary Care Trusts and 52% (102/196) of specialist paediatric units submitted data (with over 750,000 individual patient records) for the National Diabetes Audit. Data are extracted annually from the patient record systems in participating health care organisations.	Indicators 1-6

### Appraisal of data quality

As detailed in Table 5, of the 13 data sources, two (13%) were assessed as of poor quality, three (23%) as of satisfactory quality, and eight (62%) as of good quality. The poor quality of the two Australian data sources was driven by lack of representativeness in sampling and relatively low national coverage of the data.

**Table 5:** Appraisal of data quality

Country	Data source/year	Quality assessment score				
		
		Sample representativeness	National coverage of diabetes care data	Measurement objectiveness	Response rate	Overall score*
**Australia**	General Practice Divisions Information Online System 2006-07 [26]	0 Voluntary participation by GPs and general practices	1 65% (77/119) of divisions provided data	1 Clinical records	Not applicable	0.66

	National General Practice Divisions Diabetes Program, 2002 [35,36]	0 Voluntary participation by GPs and general practices	0 13% (16/120) of divisions provided data	1 Clinical records	Not applicable	0.33

	Australian National Diabetes Information Audit & Benchmarking 2006 [27]	0 Convenience sampling of one month clinical encounter data	0 27% (16/60) of Diabetes Centres provided data	1 Clinical records	Not applicable	0.33

	Health Insurance Commission General Practice Statistics, 1999-2000 [1]	1 whole population data	1 Whole population coverage	1 Insurance billing data related to laboratory tests	Not applicable	1

	The Australian Diabetes, Obesity, and Lifestyle Study (AusDiab), 1999-2000 [34,37]	1 Stratified, multi-stage sampling	1 Nation wide	0.5 Interviews Laboratory tests	1 > 60%	0.88

**Canada**	Canadian Community Health Survey 2005 [28]	1 Stratified, multi-stage sampling	0 46% (6/13) of Canadian provinces/territories provided data	0 Interviews	1 > 60%	0.50

**New Zealand**	National Get Checked Programme 2006 [29]	1 Whole population data	1 64% of estimated diabetes patients in the country	1 Clinical records	Not applicable	1

**The US**	Medical Expenditure Panel Survey (MEPS), 2004 [30]	1 Representative sample of households	1 Nation wide	0 interviews	1 > 60%	0.75

	CDC Behavioural Risk Factor Surveillance System (BRFSS) [20]	1 Representative sample of adults	1 Nation wide	0 Telephone interviews	1 > 60%	0.75

	National Health and Nutrition Examination Survey (NHANES), 1999-2004 [30]	1 Representative sample	1 Nation wide	0.5 Interviews Laboratory	1 > 60%	0.88

	Health Plan Employer Data and Information Set (HEDIS), 2006 [31]	1 Representative patient records from managed care organisations	1 90% of the US health plans provided data	1 Clinical records	Not applicable	1

	Indian Health Service, Clinical Reporting System, 2006 [32]	1 All patient records from participating health facilities	1 All 12 Indian Health Service Areas provided data	1 Clinical records	Not applicable	1

**The UK**	National Diabetes Audit 2005-2006 [33]	1 All patient records from participating primary and secondary care sectors	0 43% (131/305) of Primary Care Trust and 52% (102/196) of specialist paediatric units provided data	1 Clinical records	Not applicable	0.66

* Grading of the quality assessment score: 0-0.33 (poor); 0.34-0.66 (satisfactory); 0.67-1 (good)

### Processes of diabetes care

#### Availability of data

Annual HbA1c testing, lipid testing, kidney function examination, and eye examination rates are shown in Tables 6 and 7. Four countries (Australia, New Zealand, the US and the UK) had national data available for all of these four process measures, but Canadian data were available for only two process measures. Data sources varied between countries: Australian data were mainly derived from diabetes registers in general practice and specialist clinics; Canadian data were based on the Canadian Community Health Survey; the US data were collected through the Health Plan Employer Data and Information Set (HEDIS) using medical record audits; the UK data were from the National Diabetes Audit; and New Zealand data were from the National Get Checked Programme. Except for Canada, the main data for the other four countries are derived from health service records.

**Table 6:** Annual HbA1c and lipid testing for people with diabetes by country

Country/Targeted population	Annual HbA1c testing	Annual lipid testing	Data source
**Australia**			
Patients in general practice diabetes registers	65%	50%	General Practice Divisions Information Online System 2006-07 [26]
			
Patients visiting specialist diabetes clinics	93%	79%	Australian National Diabetes Information Audit & Benchmarking, 2006 [27]
**Canada**			
Adults ≥ 18 years living in private households	74%	-	Canadian Community Health Survey 2005 [28]
**New Zealand**			
Patients on primary care diabetes registers	64%	64%	National Get Checked Programme, 2006 [29]
Subgroups: NZ European	68%	68%	
Maori	39%	39%	
Pacific Island	99%	99%	
**The US**			
Patients ≥ 40 years old	92%	-	Medical Expenditure Panel Survey, 2004 [30]
Patients ≥ 18 years old with home telephones	61%	-	CDC Behavioural Risk Factor Surveillance System, 2001 [20]
Patients (18-75 years old) with Medicaid, Medicare, or Commercial Insurance	Medicaid: 78% Medicare: 87% Commercial: 88%	71% 85% 83%	Health Plan Employer Data and Information Set (HEDIS), 2006 [31]
American Indians and Alaska Natives	79%	60%	Indian Health Service, Clinical Reporting System, 2006 [32]
**The UK**			
Patients receiving care from primary and secondary care sectors	83%	81%	National Diabetes Audit 2005-2006 [33]

**Table 7:** Annual kidney function and eye examination for people with diabetes by country

Country/Targeted population	Annual kidney function examination	Annual eye examination	Data source
**Australia**			
General patients	-	77% (2 yrs)	The Australian Diabetes, Obesity, and Lifestyle Study [34]
Patients in GP diabetes registers	27%	32%	National Divisions Diabetes Program, 2002 [35,36]
Patients visiting specialist diabetes clinics	70%	-	Australian National Diabetes Information Audit & Benchmarking, 2006 [27]
Patients whose tests were processed by Medicare	18%	70% (2 yrs)	Health Insurance Commission General Practice Statistics, 1999-2000 [1]
**Canada**			
Adults ≥ 18 years living in private households	-	48%	Canadian Community Health Survey 2005 [28]
**New Zealand**			
Patients on primary care diabetes registers	64%	71% (2 yrs)	National Get Checked Programme, 2006 [29]
Subgroups: NZ European	68%	73% (2 yrs)	
Maori	39%	68% (2 yrs)	
Pacific Island	99%	66% (2 yrs)	
**The US**			
Patients ≥ 40 years old	-	68%	Medical Expenditure Panel Survey, 2004 [30]
Patients ≥ 18 years old with home telephones	-	67%	CDC Behavioural Risk Factor Surveillance System, 2001 [20]
Patients (18-75 years old) with Medicaid, Medicare, or Commercial Insurance	Medicaid: 75% Medicare: 85% Commercial: 80%	51% 62% 55%	Health Plan Employer Data and Information Set (HEDIS), 2006 [31]
American Indians and Alaska Natives	55%	49%	Indian Health Service, Clinical Reporting System, 2006 [32]
**The UK**			
Patients receiving care from primary and secondary care sectors	83%	61%	National Diabetes Audit 2005-2006 [33]

#### Comparison between countries

For across nation comparison, data need to be collected using similar methods and during similar time periods. Based on medical record data, the US and UK performed relatively well across the four process indicators when compared with Australia and New Zealand (Tables 6 and 7). For example, annual HbA1c testing and lipid testing were delivered to 70-80% of patients in the US and UK; the corresponding figures for Australia and New Zealand were 50-60%.

Based on data from similar population level surveys in Canada and US, the former had higher annual HbA1c testing rate but relatively lower eye examination rate when compared with the latter.

#### Sub-group comparison within countries

In Australia, annual checks for the four care processes were consistently higher for patients receiving specialist services than those cared for solely in general practice (see Tables 6 and 7). Poor identification of Indigenous status of patients in medical records limited the potential to report corresponding data for Indigenous people.

Of diabetes patients registered in New Zealand, Pacific Islanders had the highest annual HbA1c, lipid and kidney function testing rates, followed by those of European origin. Delivery of the key processes of care to Maori people was relatively low.

In the US, diabetes patients on Medicaid tended to have lower annual checking rates for the four processes compared to those on Medicare or commercial insurance. Annual lipid testing and renal function examination rates were substantially lower among American Indians and Alaska Natives than general populations.

Although the Canadian Community Health Survey collects information on Aboriginal status, the data on diabetes care among Aboriginal populations had not been reported at the time this review was completed.

### Proximal outcomes

#### Availability of data related to HbA1c and lipid control

Four countries had data available to construct indicators for HbA1c and lipid control at the national level (see Tables 8 and 9). Australia and New Zealand data were based on diabetes registers, the US used clinical audit data from the HEDIS, and the UK data were obtained through the National Diabetes Audit. Additionally, HbA1c control data could be drawn from the periodical National Health and Nutrition Examination Survey in the US, and a national population-based survey (the AusDiab) in Australia.

**Table 8:** HbA1c control for people with diabetes by country

Country/Targeted population	Criteria	Percent	Data source
**Australia**			
Patients in GP diabetes registers	HbA1c < 7.0%	57%	General Practice Divisions Information Online System 2006-07 [26]
Patients visiting specialist diabetes clinics	HbA1c < 7.0%	38%	Australian National Diabetes Information Audit & Benchmarking, 2006 [27]
General patients	HbA1c < 7.0%	57%	AusDiab 1999-2000 [37]
**Canada**	-	-	
**New Zealand**			
Patients on primary care diabetes registers	HbA1c < 8.0%	73%	National Get Checked Programme, 2006 [29]
Subgroups: NZ European		78%	
Maori		60%	
Pacific		56%	
**The US**			
Patiens≥ 40 years old	HbA1c < 7.0%	49%	National Health and Nutrition Examination Survey (NHANES), 1999-2004 [30]
Adults (18-75 years old) with Medicaid, Medicare, or Commercial Insurance	HbA1c < 7.0% Medicaid Medicare Commercial	30% 46% 42%	Health Plan Employer Data and Information Set (HEDIS), 2006 [31]
American Indians and Alaska Natives	HbA1c < 7.0%	31%	Indian Health Service, Clinical Reporting System, 2006 [32]
**The UK**			
Patients receiving care from primary and secondary care sectors	HbA1c < 6.5% HbA1c 6.5-7.5% HbA1c > 7.5%	22% 36% 42%	National Diabetes Audit 2005-2006 [33]

**Table 9:** Lipid control for people with diabetes by country

Country/Targeted population	Criteria	Percent		Data source
**Australia**				
Patients in GP diabetes registers	TC < 4 mmol/lL	44%		General Practice Divisions Information Online System 2006-07 [26]
Patients ≥25 years old	TC≥5.5 mmol/L HDL-C < 1.0 mmol/L Triglycerides > 4.0	Male 58% 22% 8%	Female 69% 24% 7%	AusDiab 1999-2000 [37]
Patients visiting specialist diabetes clinics	TC≥5.5 mmol/L LDL-C≥2.6 mmol/L	14% 82%		Australian National Diabetes Information Audit & Benchmarking, 2006 [27]
**Canada**	-	-		
**New Zealand**	-	-		
**The US**				
Patiens≥ 40 years old	TC < 5.2 mmol/L LDL-C < 2.6 mmol/L	48%		National Health and Nutrition Examination Survey (NHANES), 1999-2004 [30]
Patients (18-75 years old) with Medicaid, Medicare, or Commercial Insurance	Medicaid Medicare Commercial	31% 47% 43%		Health Plan Employer Data and Information Set (HEDIS), 2006 [31]
**The UK**				
Patients receiving care from primary and secondary care sectors	TC < 5 mmol/L	73%		National Diabetes Audit 2005-2006 [33]

TC: total cholesterol; LDL-C: low density lipoprotein cholesterol; HDL-C: high density lipoprotein cholesterol.

#### Comparison between countries

A number of different laboratory methods are used to measure the HbA1c level, so the normal ranges for HbA1c may vary between laboratories. Moreover, varying cut-points used in different countries' reports make international comparison difficult. For example, while HbA1c < 7.0% was reported for Australia and the US data, HbA1c < 8.0% was reported for New Zealand data, and cut-points of < 6.5%, 6.5%-7.5% and > 7.5% were used in the UK (Table 8). Most of the diabetes clinical guidelines [38-40] set HbA1c less than 7.0% as optimal glycaemic control.

Based on clinical data and allowing for adjustment of differences in HbA1c cut-points used, glycaemic control for patients in Australia and New Zealand appeared to be relatively better than patients in the US and UK (Table 8).

Difficulties in comparing blood lipid control were caused by the diversity of lipid parameters (total cholesterol, low density lipoprotein cholesterol, high density lipoprotein cholesterol, and triglycerides) and the varying cut-points employed (Table 9). According to the clinical guidelines from the US, New Zealand and Australia [38-41], recommended optimal lipid control for diabetes patients are as follows: total cholesterol < 4.0 mmol/L, LDL-cholesterol < 2.6 mmol/L (or 2.5), HDL-cholesterol > 1.0 mmol/L (or 1.1), and triglycerides < 1.7 mmol/L.

Using total cholesterol control among patients as an example, the UK performed relatively better, followed by Australia and the US (Table 9).

#### Sub-group comparison within countries

In Australia, patients in general practice were more likely to have optimal HbA1c control than those visiting specialist clinics (57% versus 38%). However, this preliminary comparison may not reflect true differences in the quality of care in these two different settings, as patients referred to specialist clinics tend to be those with uncontrolled diabetes or more complications. Regarding blood lipid control, patients visiting specialist diabetes clinics tended to have better total cholesterol control compared those cared for by GPs.

The proportion of New Zealand European patients with HbA1c less than 8.0% was higher than that among Maori or Pacific Islander counterparts (78% versus 56-60%).

The US data revealed that patients on Medicaid were more likely than those on Medicare or commercial insurance to have poor blood glycaemic control and an elevated LDL-cholesterol level. Control of HbA1c among American Indian and Alaska Native patients (31% with HbA1c < 7.0%) was poorer than that among general patients (range 30-46%) in the US (Table 8).

## Discussion

This internet-based review reveals that the five selected countries have various data collection systems which, to some extent, can be used to construct the OECD diabetes indicators for cross country comparison. Routine data collection mechanisms also allow countries to monitor long-term trends of diabetes care. While New Zealand and the US have relatively well developed data collection systems to assess diabetes care among indigenous populations at the national level, Australia and Canada lag behind substantially in this area and have no national level diabetes care data available for indigenous populations. Countries can learn from each other to strengthen data collection systems and to improve population-based diabetes care.

### Existing data sources for constructing OECD indicators: strengths and weaknesses

#### Diabetes registries

Australia and New Zealand have national data derived from general practice diabetes registers to monitor utilisation of diabetes services. Diabetes registers are useful vehicles for prospectively tracking patients, and data collected are by-products of delivery of care. An issue of concern is to what extent the register covers diabetes populations. The registers covered 64% of estimated diabetes patients in New Zealand in 2006. However, the corresponding coverage information is unknown in Australia. Provision of data by general practices on a voluntary basis in Australia may lead to substantial bias in estimating population level indicators. Maximising coverage of diabetes registers and participation of general practices in data collection and reporting systems should be a priority for future health information development in Australia.

#### Administrative records: insurance billing data and electronic laboratory data

Administrative insurance data have been used in Australia and are planned to be used in Canada for surveillance of diabetes services [42]. The use of insurance data has a number of advantages, including: 1) coverage of nearly the entire population, due to publicly funded health insurance arrangements in these two countries; 2) these data are not subject to recall bias; and 3) computerised databases already exist. The use of Medicare occasions of service data (1993-1997) in New South Wales (Australia) was found to be a reliable, timely and cost-efficient way to monitor health service utilisation for people with diabetes [43]. All of the four diabetes process indicators could be constructed using Medicare data at the national level for Australia [1]. To date, the use of Australian Medicare data to monitor diabetes care is on an ad hoc basis. If specific efforts are made to routinely analyse and report Medicare data at the national level, information provided for diabetes indicators will be valuable for policy makers, health providers, researchers and the public to combat and control diabetes. In the US, Medicare claims data are used by the Centres for Medicare and Medicaid Services to monitor quality of diabetes care for Medicare beneficiaries [44].

The Canadian National Diabetes Surveillance System [42] is a national standardised database linking three types of person-specific administrative data (Physician Claims File, Hospital File and Health Insurance Registry). The first report from the Canadian National Diabetes Surveillance System contained prevalence and mortality data. The 2008 report provides information on health services utilisation in terms of the number of visits to family physicians and specialists by patients [45]. Further development of the system has the potential to report national diabetes care data related to the four process indicators used in this review. Importantly, at the Canadian province level, there has been reported use of clinical and administrative data to evaluate diabetes care in terms of process indicators (e.g. in British Columbia) [46].

One of the disadvantages in using administrative insurance data is that these data include only information on those who use health services; for people with diabetes, only those with a diagnosis from a health professional are included. Also, administrative data lack detailed information on characteristics of patients such as ethnicity, which prevents related sub-group analysis. Another drawback is that insurance data do not record the test results, for example, HbA1c and total cholesterol levels.

Electronic laboratory data are becoming more common in many OECD countries. Therefore, it is possible to construct measures for HbA1c and lipid control from such data sources. The challenge is how to link these data with other data such as insurance billing records using a unique identifier for individual patients.

#### Auditing medical records

As illustrated in this review, data collection systems such as National Clinical Audit in the UK and the HEDIS in the US, which obtain data through auditing of medical records, provide all the information needed for the six OECD diabetes process and proximal outcome indicators. The quality of data from the clinical audits is generally good. With a more widespread use of electronic medical records, clinical auditing will be less labour-intensive and more feasible to provide population-based information on diabetes care.

The HEDIS data cover people enrolled in managed-care plans of different types (commercial insurance, Medicare, and Medicaid), but do not cover people without health insurance (estimated to be 45 million in the US, accounting for 16% of the whole population) [31]. A published national study sheds new light on the quality of health care (including diabetes care) delivered to adults in the US [47]. Based on reviews of medical records, the study found that adherence to the processes relating to six monthly HbA1c testing, ever-documented total cholesterol test, annual urine protein test, and annual eye examinations, were 24%, 58%, 24%, and 14%, respectively. These results were apparently lower than those reported by managed-health plans, indicating quality of diabetes care is lower in the nation as a whole than in those people with health insurance.

#### Periodical national health surveys

The Behavioural Risk Factor Surveillance System (BRFSS) in the US and the Canadian Community Health Survey collect data annually and every two years respectively, based on interviews of participants. While reporting population based diabetes care process information on a regular and ongoing basis is a major strength of those surveys, recall biases from respondents and lack of information on outcomes (such as HbA1c control) are the major weaknesses. The US National Health and Nutrition Examination Survey (NHANES) collects data using interviews and laboratory tests, lending its capacity to report HbA1c and lipid control among patients with diabetes.

In Australia, the National Health Survey (NHS) provided data for estimating prevalence of self-reported diabetes. However, it does not have diabetes-specific components in the questionnaire that allow further analysis of utilisation of diabetes services. The Australian Diabetes, Obesity, and Lifestyle Study (AusDiab) was a one-off population-based survey, ruling out its ability to monitor diabetes care over time.

Our assessment of quality of data sources included in the review showed that the majority of them (11/13) were of satisfactory or good quality. This should encourage policy makers, administrators, and service providers to continue their efforts and investments in the routine data collection systems for ongoing monitoring and improving diabetes care. The poor quality of the two data sources was largely due to poor representativeness in sampling and low national data coverage, highlighting the areas for improvement in the data collection systems.

### Strengths and limitations of the present review

We primarily searched websites of health departments and leading national organisations related to diabetes care in each of the five countries, to identify publicly released reports relevant to diabetes care. With the internet playing a significant role in access to and dissemination of information, the search strategy used was well suited to the aims of the review.

This review is subject to some limitations. Due to the evolving nature of website contents (information is added or removed from time to time), there was possibility that some relevant reports might not be captured by the internet search. We updated the search in 2009/2010 after the initial search in 2006 to minimise this limitation.

### Caveats in interpreting OECD diabetes indicators

#### Tensions between diabetes performance indicators and clinical practice guidelines

Diabetes clinical guidelines are intended to set optimal standards of care for individual patients by accounting for age and severity of the disease. Unlike clinical guidelines, performance indicators focus on measuring quality of care at a population level and creating a basis for accountability and quality improvement in the health care system [48]. In patients with a variety of disease states, comparison of care across healthcare systems becomes difficult if important confounders (e.g. patients' age and health status) are not adjusted for.

For the above-mentioned reasons, performance indicators reflect a level of care which is less than the ideal standard, to facilitate comparison across populations without a need for risk adjustment [48]. For example, with regard to frequency of HbA1c testing, the American Diabetes Association's guidelines recommend that HbA1c testing be performed at least once every six months for people with stable blood glucose levels and every three months in those whose therapy has changed or who are not meeting blood glucose goals [38]; performance indicators, however, only measure whether HbA1c has been tested at least once in the last 12 months.

While clinical guidelines play an essential role in promoting best quality of care for individuals, performance indicators have been primarily employed in an attempt to reflect quality of care at the population level in a convenient and feasible way. It is important to avoid interpretation of indicators as definitions of the "standard of care".

#### Blood pressure control and foot examination: forgotten areas

The current proposed OECD indicators do not cover services relating to blood pressure control and foot examinations, as these indicators are perceived as not feasible to collect from administrative data sources.

However, in this review, it was found that the most reliable and comprehensive data for current use were obtained from auditing medical records. It is possible to extract data on blood pressure control and foot examinations through this approach.

Therefore, inclusion of blood pressure control and foot examinations in the spectrum of OECD indicators would provide a holistic profile regarding quality of diabetes care. Exclusion of these indicators may risk underestimating their importance in clinical practice and discourage efforts to collect relevant data.

#### Treatment for patients with diabetes

It is notable that treatment of diabetes has not been directly monitored by OECD indicators, largely due to complexity in data collection. Treatment is tailored for individuals depending on the patient's disease status, and plays a crucial role in diabetes management. Clinical trials have found that a one percentage point reduction in HbA1c levels would reduce micro-vascular complications by 25% to 30% [49,50] and a 10 mmHg reduction in blood pressure would decrease macro- and micro-vascular complications and death rates by 32% [51]. Improved control of blood lipids can reduce risk of coronary heart disease by 39% and risk of death by 43% [52].

RAND's Quality Assessment Tools System offers indicators relating to diabetes treatment [53], and application of these indicators in a national study in the US has provided insight into adherence to recommended treatment regimens. For people with newly diagnosed diabetes 56% received dietary and exercise counselling. In type 2 diabetes patients, use of oral hypoglycaemic agents for those inadequately controlled on dietary therapy was 38% and use of insulin for those inadequately controlled on oral hypoglycaemics was 39%. Fifty-five percent of diabetics were offered an ACE inhibitor within 3 months of the notation of proteinuria unless contraindicated.

The AusDiab study reported the treatment pattern among Australian adults with type 2 diabetes [37]. While 32% of diabetes patients were on diet regimen only, 58% used oral hypoglycaemic agents and diet only, and another 10% took insulin. Bailie and colleagues reported pharmaceutical interventions for diabetes patients in remote Aboriginal communities in the Northern Territory [54]. During the 3-years study period, 75-79% of Aboriginal patients took oral hypoglycaemic agents, and 4-7% used insulin.

### Comparison of diabetes care and health system performance across countries

Allowing for the difference in data collection methods, there was relatively wide variation in performance related to processes of diabetes care across the five countries. For example, annual HbA1c testing rates were 64-65% in New Zealand and Australia; the corresponding rate for the UK was 83%. Relatively better performance in such indicators in the UK general practice may be partly due to payment based on capitation, which requires general practitioners to clearly define a population and take a population-based approach to clinical care [55]. In contrast, most general practitioners in Australia are paid on a fee-for-service basis, with no clearly defined populations for service delivery.

However, relatively less variation was observed in relation to proximal outcomes such as HbA1c control across the countries. To achieve HbA1c control targets as set by clinical guidelines is much more complicated than to simply test HbA1c levels. Previous studies comparing primary care and health system performance in these five countries have identified common deficiencies in health care systems, including poor care coordination and deficient patient-doctor communication [56,57]. These can result in duplicate tests, delays in care, failure of doctors to discuss care goals and options with patients and to review medication regimens. Such widespread deficiencies across the five countries are likely to contribute to the suboptimal control of HbA1c among patients.

In a recently published OECD Health at a Glance 2009 report [58], two diabetes-related indicators [admission rates for acute diabetic complications; diabetes lower extremity amputation rates (denominators were general populations rather than peoples with diabetes as specified in the original indicator set listed in Table 3)], along with the other 21 indicators, have been used to measure health system performance across OECD countries. Diabetes lower extremity amputation rates were higher in the US (36/100,000 population) than in New Zealand, Canada and the UK (9-12/100,000), as were admission rates for acute diabetic complications (57/100,000 versus 1-32/100,000). It appeared that diabetes management might be much poorer at the primary care level in the US which led to substantially higher rates of diabetes-related complications. Our review provided evidence of relatively poor control of HbA1c among patients in the US, a direct measure of diabetes management in primary care. With availability and comparability of data up to standard in the future, inclusion of indicators such as HbA1c testing and control into selected OECD health care quality indicators for reporting would enhance understanding of variation in health system performance across countries and point to ways for improvement.

### Implications for practice and policy

Measured by OECD diabetes indicators, considerable gaps were revealed in the five selected countries in relation to both optimal diabetes care and availability/quality of data. Countries can learn from each other to strengthen these areas.

For Australia, one of the priorities is to strengthen routine data collection and reporting systems using diabetes registers in general practice, and to improve the coverage of the diabetes registers.

In line with the national funding plan to implement "Strategies for the prevention and control of diabetes in New Zealand", specific funding has been allocated to Maori and Pacific Island people for their own initiatives, including annual free checks and treatment. This designated financial arrangement increased access to high quality diabetes care for Maori and Pacific peoples as well as availability of information. Further implementation of the programme in New Zealand should increase its reach to indigenous and non-indigenous populations in the country. The national diabetes clinical audit system in the UK proves to be a feasible and efficient information system in monitoring diabetes care.

In the US, the most comprehensive and timely data are reported by the National Committee for Quality Assurance to measure performance of managed care plans through the HEDIS. The US Indian Health Service has a well developed Clinical Reporting System to routinely support diabetes care data for American Indians and Alaska Natives. However, for Canada, performance in most key indicators is currently unknown at the national level. Further development of the Canadian National Diabetes Surveillance System might serve as a vehicle to narrow the current information gap.

There is an urgent need for diabetes care information among indigenous populations in Australia and Canada. The process and proximal indicators provide information to inform efforts for prevention and early detection of diabetes-related complications. However, the evidence in this review is especially deficient in these areas for indigenous populations in Australia and Canada. Based on New Zealand and US experiences, with specific commitment to data collection for indigenous populations through effective policy and legislation, it is possible to obtain parallel information for these populations in relation to key indicators of diabetes care.

## Conclusions

Substantial efforts have been made in the five countries to develop routine data collection systems to monitor performance of diabetes management. Available performance data identify considerable gaps in clinical care of diabetes across countries. Policy makers and health service providers across countries can learn from each other to improve data collection and delivery of diabetes care at the population level.

## Competing interests

The authors declare that they have no competing interests.

## Authors' contributions

DS conceived and designed the review, conducted literature search and synthesis of data, and drafted the manuscript. RB and TW contributed to conceptualisation and design of the review, interpretation of results, and revision of the manuscript. ZW contributed to data abstraction and synthesis, interpretation of results and manuscript revision. All authors contributed to, have read and approved the final manuscript.

## Pre-publication history

The pre-publication history for this paper can be accessed here:

http://www.biomedcentral.com/1472-6963/10/169/prepub

## Supplementary Material

Additional File 1 **Quality assessment checklist for observational studies/surveys/evaluations**. The file contains an assessment tool used in this study to appraise the quality of data included. An overall score is calculated to classify the data quality into three categories: poor (score 0-0.33), satisfactory (0.34-0.66), or good (0.67-1.00). Click here for file
