# Supplementary material for: Comparison of diabetes management in five countries for general and indigenous populations: an internet-based review
Source: BMC Health Serv Res. 2010 Jun 17;10:169. doi: 10.1186/1472-6963-10-169 (PMC2903584; doi:10.1186/1472-6963-10-169)
Supplement: Additional File 1 — Quality assessment checklist for observational studies/surveys/evaluations. The file contains an assessment tool used in this study to appraise the quality of data included. An overall score is calculated to classify the data quality into three categories: poor (score 0-0.33), satisfactory (0.34-0.66), or good (0.67-1.00). [file 1472-6963-10-169-S1.DOC]

**Additional File 1: Quality assessment checklist for observational studies/surveys/evaluations**

1. Was the sampling method representative of the population intended to the study?

| A. | Non-probability sampling (including: purposive, quota , convenience and snowball sampling) | 0 |
| --- | --- | --- |
| B. | Probability sampling (including: simple random, systematic, stratified g, cluster, two-stage and multi-stage sampling) | 1  1 |
| C. | The whole population included |  |

1. National coverage of data collected (e.g. the proportion of provinces/states/regions/organisations covered)

| A. | <50% | 0 |
| --- | --- | --- |
| B. | ≥50% | 1 |
|  |  |  |

1. Were the measurements objective?

| A. | By questionnaires (self-reported) | 0 |
| --- | --- | --- |
| B. | By clinical records or laboratory tests | 1 |
| C. | Combination of A and B | 0.5 |

1. Did the study report any response rate? (If the reported response rate is below 60%, the question should be answered “No”.)

0

| A. | No or response rate<60% |  |
| --- | --- | --- |
| B.  C. | Response rate≥60%  Not applicable | 1  n/a |
|  |  |  |

Scoring method: Total score divided by total number of all applicable items

Grading of the overall quality assessment score:

| 0 – 0.33 | 0.34 – 0.66 | 0.67 - 1 |
| --- | --- | --- |
| poor | Satisfactory | Good |
